# Supplementary material for: Dynamic transcriptomic profiles of zebrafish gills in response to zinc supplementation
Source: BMC Genomics. 2010 Oct 11;11:553. doi: 10.1186/1471-2164-11-553 (PMC3091702; doi:10.1186/1471-2164-11-553)
Supplement: Additional file 2 — Interactive Direct Interaction Network representing the molecular interactions between zinc, copper, iron, calcium and proteins encoded by transcripts changed by zinc supplementation. Mini web-site containing index.html and hyperlinked pages in subdirectory describing a Direct Interaction Network automatically generated based on curated interactions contained within the proprietary PathwayArchitect database. Ovals represent proteins and the circles symbolize metal ions. Objects are coloured by their abundance in zebrafish at the time-point they were significantly different from the control is a scale from -4 fold (dark green) to +4 fold (dark red). Where significant differences were found at more than one time-point, the colour overlay shows expression at the first instance. Dark blue squares denote 'binding', and light blue squares 'expression'; green squares stand for 'regulation', green diamonds for 'metabolism', and green circles for 'promoter binding'. Arrow heads indicate directionality of the interaction where annotated. All nodes and edges can be further interrogated by selecting the relative area of the image. [file 1471-2164-11-553-S2.zip › PathwayArchitect Zn xs DIN/460290.html]

# REGULATION:

|  |  |
| --- | --- |
| Type | REGULATION |
| Effect | Positive |


---

|  |  |
| --- | --- |
| Score | 0 |


---

|  |  |
| --- | --- |
| Reference Count | 10 |


---

|  |  |
| --- | --- |
| Mechanism | Unknown |


---

|  |  |
| --- | --- |
| Reference:0 || Sentence | "Ethanol administration counteracts the protective effect of copper on iron concentration, total and latent iron binding capacity, transferrin saturation index and ceruloplasmin serum content." |
| PMID | 2616386 |
| Year | 1989 |
| Species | Rat |
| Journal | Pol J Pharmacol Pharm |
| RefScore | 0 |
| Source | PArchNLP |
  |
|  |  |


---

|  |  |
| --- | --- |
 Reference:1 || Sentence | "When the exposures were analyzed as continuous variables, the adjusted RRs (CI) were 1.66 (1.03-2.68) for 100 microg/dl iron increase, 1.17 (1.01-1.36) for 10% TS increase, 1.98 (1.12-3.50) for 100 microg/dl copper increase, and 0.57 (0.16-1.96) for 100 microg/dl zinc increase." |
| PMID | 15036223 |
| Year | 2004 |
| Species | Human |
| Journal | Ann Epidemiol |
| RefScore | 1 |
| Source | PArchNLP |
  ||  |  |


---

|  |  |
| --- | --- |
 Reference:2 || Sentence | "A model is proposed wherein copper promotes iron depletion of intestinal Caco-2 cells, creating a deficiency state that induces upregulation of iron transport factors." |
| PMID | 11842003 |
| Year | 2002 |
| Species | Human |
| Journal | Am J Physiol Gastrointest Liver Physiol |
| RefScore | 1 |
| Source | PArchNLP |
  ||  |  |


---

|  |  |
| --- | --- |
 Reference:3 || Sentence | "Interestingly, exposure to copper increased iron efflux from Caco-2 cells and up regulated IREG1 (iron-regulated mRNA) expression." |
| PMID | 12220667 |
| Year | 2002 |
| Species | Human |
| Journal | FEBS Lett |
| RefScore | 1 |
| Source | PArchNLP |
  ||  |  |


---

|  |  |
| --- | --- |
 Reference:4 || Sentence | "The influence of copper supplementation on iron uptake and storage was examined in a cell line capable of hemoglobin synthesis." |
| PMID | 1508944 |
| Year | 1992 |
| Species | Human |
| Journal | Proc Soc Exp Biol Med |
| RefScore | 1 |
| Source | PArchNLP |
  ||  |  |


---

|  |  |
| --- | --- |
 Reference:5 || Sentence | "The results demonstrated that copper supplementation of human K562 cells was associated with higher cytosolic iron levels and ferritin levels." |
| PMID | 1508944 |
| Year | 1992 |
| Species | Human |
| Journal | Proc Soc Exp Biol Med |
| RefScore | 3 |
| Source | PArchNLP |
  ||  |  |


---

|  |  |
| --- | --- |
 Reference:6 || Sentence | "Copper supplementation of the cell culture altered the initial rate of iron uptake from transferrin and enhanced iron uptake in noninduced cells; however, in hemin-induced K562 cells, which express fewer transferrin receptors on the cell surface, copper appeared to reduce iron uptake." |
| PMID | 1508944 |
| Year | 1992 |
| Species | Human |
| Journal | Proc Soc Exp Biol Med |
| RefScore | 1 |
| Source | PArchNLP |
  ||  |  |


---

|  |  |
| --- | --- |
 Reference:7 || Sentence | "Copper may interfere with iron absorption by binding to mucosal transferrin." |
| PMID | 7447387 |
| Year | 1980 |
| Species | Rat |
|  | Human |
| Journal | Ann Clin Lab Sci |
| RefScore | 0 |
| Source | PArchNLP |
  ||  |  |


---

|  |  |
| --- | --- |
 Reference:8 || Sentence | Compared to microscale Fe(0) (<100mesh), the efficiency and rate of nitrate removal using uncatalyzed and catalyzed nano-Fe(0) were highly promoted, in which the maximum promoted rate was obtained using copper-catalyzed nano-Fe(0) (nano-Cu/Fe). |
| Year | 2005 |
| PMID | 16081210 |
| Journal | J Hazard Mater |
| RefScore | 0 |
| Source | PArchNLP |
  ||  |  |


---

|  |  |
| --- | --- |
 Reference:9 || Sentence | Compared to microscale Fe(0) (<100 mesh), the efficiency and rate of nitrate removal using uncatalyzed and catalyzed nano-Fe(0) were highly promoted, in which the maximum promoted rate was obtained using copper-catalyzed nano-Fe(0) (nano-Cu/Fe). |
| Year | 2005 |
| PMID | 16081210 |
| Journal | J Hazard Mater |
| RefScore | 0 |
| Source | PArchNLP |
  |


---

|  |  |
| --- | --- |
